# The role of agency in the implementation of Isoniazid Preventive Therapy (IPT): Lessons from *oMakoti* in uMgungundlovu District, South Africa

**DOI:** 10.1371/journal.pone.0193571

**Published:** 2018-03-07

**Authors:** Jody Boffa, Maria Mayan, Sithembile Ndlovu, Tsholofelo Mhlaba, Tyler Williamson, Reginald Sauve, Dina Fisher

**Affiliations:** 1 Department of Community Health Sciences, University of Calgary, Calgary, Alberta, Canada; 2 Desmond Tutu Tuberculosis Centre, Stellenbosch University, Cape Town, Western Cape, South Africa; 3 Community University Partnerships, Faculty of Extension, University of Alberta, Edmonton, Alberta, Canada; 4 *izImbali Zesizwe*, Pietermaritzburg, KwaZulu-Natal, South Africa; 5 Division of Public Health Medicine, University of KwaZulu-Natal, Durban, KwaZulu-Natal, South Africa; 6 Department of Paediatrics, University of Calgary, Calgary, Alberta, Canada; 7 Department of Medicine, University of Calgary, Calgary, Alberta, Canada; The Ohio State University, UNITED STATES

## Abstract

**Introduction:**

In response to revisions in global and national policy in 2011, six-month isoniazid preventive therapy (IPT) became freely available as a preventive measure for people living with HIV in the uMgungundlovu District of KwaZulu-Natal province, South Africa. Given a difference in uptake and completion by sex, we sought to explore the reasons why Zulu women were more likely to accept and complete IPT compared to men in an effort to inform future implementation.

**Methods:**

Utilising a community-based participatory research approach and ethnographic methods, we undertook 17 individual and group interviews, and met regularly with grassroots community advisory teams in three Zulu communities located in uMgungundlovu District between March 2012–December 2016.

**Findings & discussion:**

Three categories described women’s willingness to initiate IPT: women are caregivers, women are obedient, and appearance is important. The findings suggest that the success of IPT implementation amongst clinic-utilising women of uMgungundlovu is related to the cultural gender norms of *uMakoti*, isiZulu for “the bride” or “the wife.” We invoke the cultural concept of *inhlonipho*, meaning “to show respect,” to discuss how the cultural values of *uMakoti* may conflict with biomedical expectations of adherence. Such conflict can result in misinterpretations by healthcare providers or patients, and lead some patients to fear the repercussions of asking questions or contemplating discontinuation with the provider, preferring instead to appear obedient. We propose a shift in emphasis from adherence-focussed strategies, characteristic of the current biomedical approach, to practices that promote patient agency in an effort to offer IPT more appropriately.

**Implications:**

Building on existing tools, namely the harm reduction model and the use of mini-ethnography, we provide guidance on how to support women to participate as agents in the decision to initiate or continue IPT, decisions which may also impact the health and choices of the family.

## Introduction

Tuberculosis (TB) is now recognised as the deadliest infectious disease globally [1]. Immune suppression from HIV is the single strongest determinant for developing TB disease [2,3]. People living with HIV (PLWH) are 20 to 30 times more likely to develop active TB disease [4] and progress to TB disease about six times faster [5] than people without HIV. In 2015, 26% of people diagnosed with TB and 71% of people diagnosed with TB-HIV were reported on the African continent [4]. South Africa has the third highest TB incidence rate and highest TB-HIV co-infection rate globally (860 and 520 per 100 000 population, respectively [6,7]). The World Health Organisation (WHO) has identified three priority targets for reducing the burden of TB in high-incidence settings, namely, intensified case finding, shorter treatment regimens, and TB preventive therapy [3,8]. In order to provide TB preventive therapy to those at highest risk of developing TB disease, isoniazid preventative therapy (IPT) was introduced at no cost to PLWH in late 2010 [9].

Isoniazid is a medication used in combination with three other drugs to treat TB disease. Due to its low cost, tolerability, and ability to sterilise dormant bacteria, isoniazid has also been used pre-emptively to prevent TB among the latently infected. As a prophylactic, IPT is generally given as a six, nine, 12 or 36-month regimen dosed as a single 300mg daily tablet. While data from low-incidence settings suggested that IPT provides long-term protection from TB [10], the same was unclear in high TB and TB-HIV burden settings due to greater TB exposure and risk of re-infection [11].

The widespread implementation of IPT followed changes to South African and WHO guidelines in which, for the first time, a negative symptoms screen (lack of current cough, fever, night sweats, and weight loss) could be used in place of a chest radiograph to rule out active TB disease and determine eligibility for IPT [9,12]. Additionally, the use of a Tuberculin Skin Test (TST) to better target IPT was downgraded from requirement to recommendation to encourage initiation of PLWH on IPT in low-income settings where use of TST would prevent implementation–TST is a two-step process requiring a time-sensitive follow-up visit and a skilled reading of the result. These changes enabled IPT to be offered more broadly, and in 2011 uMgungundlovu District of KwaZulu-Natal province implemented the programme, offering TST-untargeted, 6-month IPT community-wide to all PLWH based upon a negative symptoms screen. Given the shift from treatment of TB disease to treatment of latent TB infection and early pilot work that found no isiZulu language equivalent for latent TB infection [13], we collected data during early implementation to assess the acceptability of IPT in this setting.

Longitudinal analysis of patient data from a community health centre in peri-urban uMgungundlovu District showed that among those completing IPT in this context, TB incidence rates declined by roughly 3 000 per 100 000 person years compared to those with no intervention, performing better than antiretroviral therapy (ART) alone and increasing the protective effect of ART when provided together [14]. Although males and females appeared to benefit similarly in terms of case reduction, the majority of PLWH who started IPT were female (72%), and females were also more likely to complete IPT compared to males (66% versus 58% respectively, p = 0.006) [15]. Given the difference in uptake and completion by sex, we utilised qualitative data to explore the reasons why Zulu women were more likely to accept and complete IPT compared to men, identifying lessons for future implementation of TB preventive therapy in similar settings.

## Methods

### Study design

#### The INH study

Utilising a community-based participatory research (CBPR) approach, we explored patient experiences and community perceptions around IPT implementation while conducting a parallel cohort study on effectiveness of TST-untargeted 6-month IPT for PLWH in uMgungundlovu District. CBPR includes a set of practices for engaging with communities affected by or complicit in the focus of the research, and involves community collaboration in the development, collection, interpretation and dissemination of the research, with an emphasis on multi-directional knowledge exchange and actionable outcomes. Utilising CBPR best practices described in Minkler and Wallerstein, we held quarterly advisory team meetings in communities to develop and undertake the research and to discuss and validate findings [16].

#### The current study

**Fig 1:**
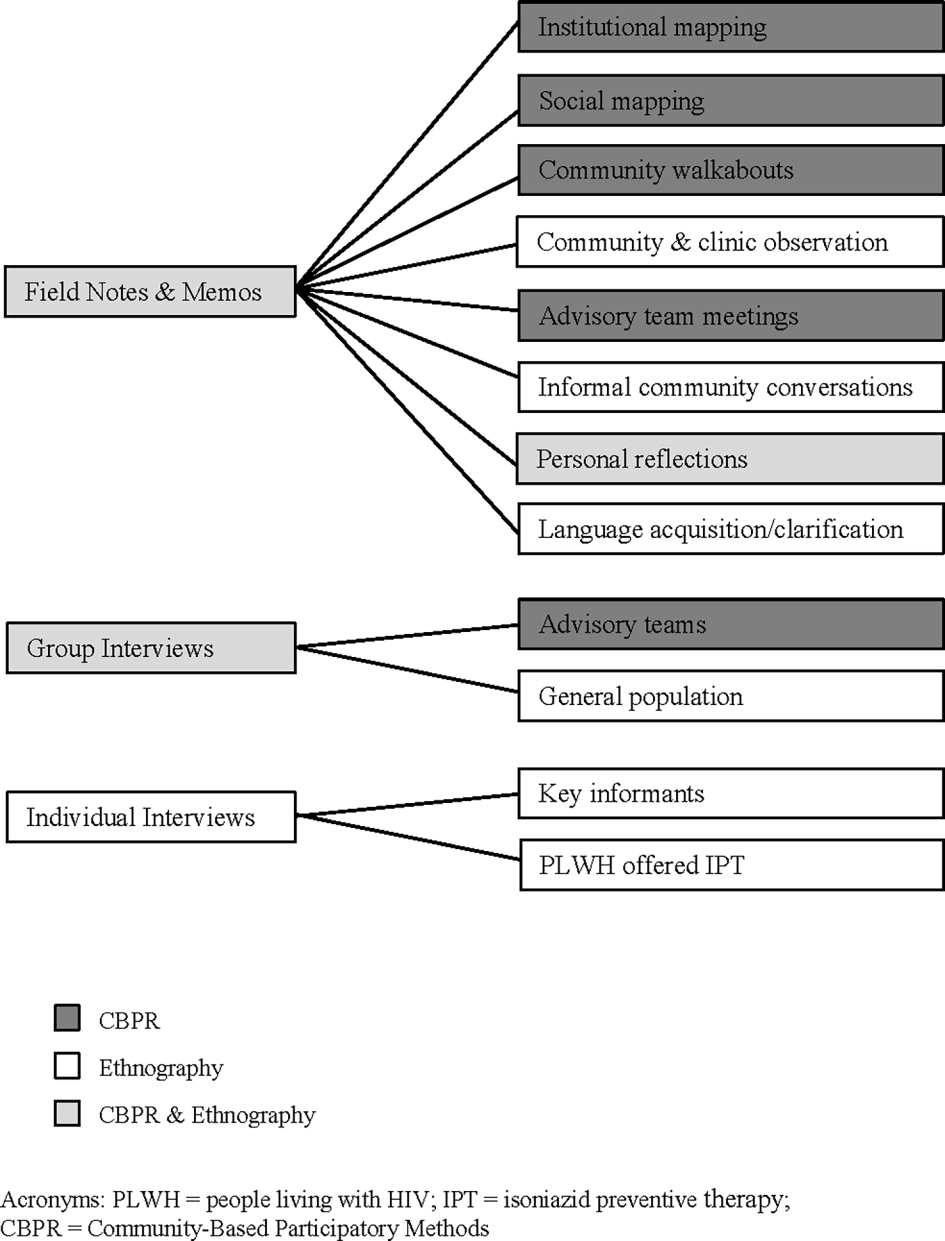
Sources of data by method of enquiry.

Led by a PhD student (JB) and community liaison (SN), we employed ethnographic methods rooted in the discipline of anthropology to study IPT implementation. Ethnography involves researchers spending extensive time in the field participating in local activities, acquiring language proficiency, and working with key informants to understand the local context and lived experience regarding the given phenomena [17,18]. These practices helped introduce investigators to local cultural norms and normalize their presence in communities. See Fig 1 for a complete list of data sources by method.

### Setting

uMgungundlovu District is located in central KwaZulu-Natal province. With a population of approximately one million people, the District reports 40% unemployment [19] with 112 000 people living on less than US$1 per day [20]. The District has an ante-natal HIV prevalence of 40.7% [19], and a TB incidence rate of 894 per 100 000 population [21,22]. Tuberculosis is the leading cause of death and of years of life lost in the District [19]. Eighty-four percent of the population does not have private healthcare insurance [19]. During the period of study, approximately 30% of the District was under 15 years of age and 65% were between the ages of 15 and 65 years of age, and there were 92 males for every 100 females [22]. Eight-five percent of the District is of African descent, among which 76.4% report isiZulu as their home language [20].

### Recruitment and sampling

In 2012 we selected three from a total of 18 communities in the Edendale Hospital catchment area in which to begin our community engagement. Prior to selection, communities were divided into groups depending upon the distance of their publically serviced clinics from Edendale Hospital based on the hypothesis that perceptions and experiences may differ between groups due to variations in proximity to health services. Peri-urban communities were defined as those in which the health clinic was ≤25 km from Edendale Hospital, rural communities were >25 km from the hospital and accessible by tar road; and remote communities were >25 km from Edendale Hospital with clinics that lacked tar road access. As we were unfamiliar with the full range of clinics in the catchment, we used a random number table to select one community in each of the three distance categories. To learn about local perspectives on IPT implementation, we interacted regularly with the selected communities between March 2012–December 2016. We purposively selected key stakeholders, and used a mix of purposive and convenience sampling to identify potential group interviewees and individuals who were offered IPT. We utilised CBPR techniques including guided walk-abouts and institutional and social mapping exercises to identify community advisory team members, key informants, and target populations for group interviews [23]. Additional key informants were recruited through ongoing community interactions. Participants for group and individual interviews were recruited via phone by a clinic nurse or at their homes by community caregivers (CCGs). CCGs are government or NGO-employed support workers who come from the communities in which they work. In the KwaZulu-Natal context, CCGs undertook home visits to care for acute or chronically ill patients. If individuals expressed interested in participating, the recruiting nurse or CCG would connect these potential participants to SN and JB to follow up with further explanation of the study and the informed consent process.

### Data collection

Data collection included ethnographic observation at clinics, public meetings, and community ceremonies; formal and informal conversation with key informants; and group and individual interviews.

From October 2014 to May 2015, we facilitated eight group interviews to learn about community perceptions about TB disease, infection, and IPT, and nine individual interviews to learn about experiences and decision-making strategies of people offered IPT. Field notes and memos from observation and informal interviews helped to articulate and develop categories. The semi-structured group and individual interview tools were reviewed and revised in collaboration with community advisory teams. Additionally, these tools were pilot tested with volunteer participants from an ART clinic in a non-selected Edendale Hospital catchment community. Individual and group interviews were digitally recorded with permission from participants. CCGs or other health workers were not present during data collection to encourage an open account of perceptions and experience.

#### Group interviews

With input from advisory teams, SN led a series of eight group interviews in the local language (isiZulu) in the three communities, while JB observed and took interview notes. Each group interview included 4–9 participants and followed established practices [24]. Questions were primarily descriptive with regard to understandings and perceptions of TB, latent TB infection, and IPT (See S1 File). Recordings were transcribed in isiZulu and then translated into English by a trained transcriber/translator. Unclear translations were reviewed between researcher and translator and interview recordings revisited by an independent translator blinded to the original translation in order to consider alternate meanings where appropriate.

#### Individual interviews

As a second stage, we investigated the individual-level experiences of PLWH offered IPT in client homes. Individual interviews took place in isiZulu with real-time English translation. English translations were transcribed for analysis and isiZulu recordings revisited by an independent translator to explore alternate meanings of words and phrases relevant to the findings [25]. Questions were primarily descriptive and structural in nature (See S2 File).

### Analysis

Individual and group transcripts, field notes, and reflections were analysed using qualitative content analysis as described by Mayan [26]. Data were organized, coded, and categorised iteratively by JB utilising Nvivo 10 software for Mac. To ensure scientific rigour, we employed several mechanisms. Data collection and analysis were dynamic and concurrent as a means of constructive validation [27], and community advisory teams and key informants were consulted throughout the process to ensure accurate descriptions of cultural concepts [28]. Negative or deviant examples were included in the sample [27, 29]. Categories were member checked by a sample of participants and peer checked with healthcare providers working in South Africa [29]. Theories were developed in relation to the data and compared to existing theory [27], a process which was also negotiated with and approved by community advisory teams [16].

### Ethics

As part of the CBPR process, we developed community partnerships with traditional leaders (*iziNkosi*), their tribal authorities, and community-tribal liaisons (*iziNduna*). Community approvals were obtained from tribal authorities and ward counsels, and institutional approvals were sought from clinic managers. Administrative approvals were granted by the KwaZulu-Natal Department of Health through the National Health Research Database system and by the uMgungundlovu District of Health. We sought and received ethical approvals for observation and group interviews from the University of Alberta’s Research Ethics Office and for patient interviews from the University of Calgary’s Conjoint Health Research Ethics Board. Additionally, the University of KwaZulu-Natal’s Biomedical Research Ethics Board granted ethics approval for both stages of the investigation.

## Findings

Data were first considered by location category (peri-urban/rural/remote); however, data codes overlapped substantially, and thus data were analysed collectively.

### Category 1: Women are caregivers

Participants overwhelmingly described caregiving as the most important role of women in the community. Understanding the composition of the typical household is helpful to illustrate this finding. Multi-generational households were common, often arranged along patrilineal lines. Architecturally the household may be made up of a central house or rondavel (round house) where family members come together to eat or socialise, with one or more ‘outbuildings’ or rondavels separating family units. In these enclaves, it was common for young wives to care for children and older family members. Participants reflected that sisters, mothers, and female in-laws were often the first point of care, especially when household members were sick: “If there is someone sick, most of the time we say maybe it’s my sister or mother who usually takes care of them because they are following the way of life that is given” (Participant number [P]6, Group Interview number [GI]4). Another participant, referring to her role said, “here in the family, I was the one who was taking care of [my mother-in-law] and my sister-in-law… and my husband as well… Even if I feel that I can’t do it, I have to do it because I am the only [healthy female]” (Individual Interview number [II]6).

Some participants described that it was seen as inappropriate for a man to provide care, for example:

Let’s say you have TB. Sometimes TB gives you swollen feet. They become so swollen that you can’t even go to the toilet and you need help to pee. Maybe sometimes you need a foot rub. You can’t ask the father; a male figure cannot take you to the toilet or rub your feet. (P3, GI8)

Key informants explained that it is often considered taboo or invasive for a male to help a female with such tasks, even among members of the same household; however, a female helping a male is considered culturally acceptable because women are thought to have more “patience” and be more “hands on” compared to their male counterparts (P6, GI4).

Women also held the overwhelming majority of caregiving positions in the formal healthcare system. Primary care centres were commonly run and staffed by female nurses called “sisters,” lay counsellors, and clinic assistants. CCGs were exclusively women. Participants described CCGs as a support system for those who lived alone or in male-only households: “there are people who are hired at hospitals who can give help if I am single and sick and live alone,” (P1, GI4). Others described CCGs as mediators between health workers and the sick: “the healthy will sometimes struggle and the doctors will work with the community caregivers to take care of the sick person” (P5, GI1). Some described how CCGs provided more than medical support: “even though I was abandoned by my husband, I did survive. The CCGs brought me some clothes, their own things, even from their own families” (II7).

### Category 2: Women are obedient

In study communities, women demonstrated obedience through several mechanisms. Obedience was demonstrated in the home through service to husbands, uncles, elders, and in-laws. For example, when meals were served, women or older female children would dish up first to men and boys and cater to their requests before looking after themselves or other females in the household. Children were often commandeered to run errands for older family members, and girls were guided from a young age to respond to the needs of the household:

We as females are being taught that we should wake up early, do this and that, and being a female you can’t go to bed before everyone. You need to make sure that even your parents are asleep first. Are the windows closed? Is the house clean? …I think not in a negative way, but it is how we’ve grown up. (Key Informant Interview number 12)

Obedience was also demonstrated through docility. Regardless of personal dispositions, women grew increasingly quiet and submissive in the presence of men and authority figures. For example, one female key informant who regularly provided insightful political context to the female research team fell silent on the subject when similar opinions were elicited in the presence of her son-in-law or other male relatives.

Obedience was also displayed more formally through appearance and body language. In the following field note, one researcher described her introduction to the importance of ritualised body language during a visit to *iNkosi* (the traditional leader) of a community:

I noted that [SN] wrapped her hair when she learned that we would meet with *iNkosi*. She later explained that women cover their hair as a sign of respect. When he entered [the rondavel] we had to bow our heads slightly, and we (the women) were to remain seated while the men stood up to recognise his entry. SN kept her head slightly bowed and eyes averted as she spoke… She explained to me later that in Zulu culture, a woman must avert her eyes in the presence of authority… She explained that it’s not always easy to navigate [what constitutes authority], but that it includes… *iNduna*, *iNkosi*, and elderly in-laws, …[although] it depends on [the personality and one’s familiarity with] the person [of authority]. (Field Notes)

During such interactions, it was common for women not to speak until invited, and even then, to keep matters short and eyes averted.

Such displays of obedience were also present in the clinic environment. Silence and submissive body language were commonly observed in clinic consultations, and female participants explained that they had reservations about appearing overly vocal in clinic encounters. One female participant explained that even when she did speak up, she was not provided an explanation and described herself as needing to be “unbearable”:

Some are called on a list and they get [IPT], but me, I don’t get it…why don’t I ever get it? …I asked and I never did get an explanation for who is given [IPT] …I told myself that maybe it’s because I’m already in [an antiretroviral] treatment programme and maybe those who have started treatment don’t get it. …you keep asking about it every time you come for follow-up and you become unbearable. (P3, GI5)

Another participant echoed her sentiment, explaining that one becomes unbearable when “you are asking irritating questions,” suggesting that, “you know too much now” (P5, GI5). As the group interview continued, other participants proposed medical reasons that might explain her ineligibility, trying to ease her anxiety. One by one, she discounted their suggestions, indicating that her tests showed she had strong liver and kidney function, for example. Later in the discussion she described another encounter that evoked deeper fears around “knowing too much.”

There was this one girl who was taking TB treatment with Nevirapine, and she asked the nurses about it because she knew she wasn’t supposed to mix them. I asked her, ‘why are you taking these?’ because I know that TB pills and Nevirapine don’t mix. She said, ‘the nurses will not even book a doctor for me.’ She became sick and she died. That killed me inside when I found out she died. I had thought about asking about her at the clinic to see if she was doing better, but I worried that [the nurses] would tell me that I know too much, just like that girl. (P3, GI5)

In another encounter, a participant described a comfortable relationship with her regular healthcare provider; however, when her physician offered her a prescription for food parcels to help ensure she had food to take with her IPT, she was unsure of what to do with it. Rather than ask him, she approached the research team to help her navigate the system on her behalf (II4).

For many participants, obedience to healthcare providers was also implied when the decision to initiate IPT was relinquished to the healthcare provider. Several participants reflected that they started IPT “because the nurses told me to” (P5, GI5) or “the results showed that I should be enrolled” (II8). Interestingly, one participant felt unsettled at the end of an interview and asked the interviewer if she ought to have had questions for her healthcare provider when she was initiated on IPT: “The only confusing part now is that maybe I was supposed to ask some questions about this pill” (II8).

Not all participants fit into this category. Although few in number, some participants took noticeable pride when speaking of the open communication they shared with healthcare providers. For example, one participant reflected that she would have probably died if not for the “tough” nurses and dedicated lay counsellors who inspired her to keep living when her husband and his family rejected her following her HIV diagnosis at the time of her first pregnancy. She explained that among clinic staff, “I’m well known. They know about my background, my health background” (II7). She later explained that this close relationship encouraged her to initiate conversations and engage more with community members and clinic staff in matters of health. Another participant whose experience did not fit with this category openly declined IPT because she had recently completed TB treatment: “I rejected it because I said ‘I am already having prevention because I have been on TB treatment, so I’m safe now from TB infection’” (II1). When asked how the healthcare providers responded to her decision, she said they thought she was clever for recognising that isoniazid was also part of her treatment regimen.

### Category 3: Appearance is important

A third category commonly reflected in the data was the importance of appearance among Zulu women. Participants spoke about cultural expectations of a woman’s outward appearance (*e*.*g*. freshly bathed, wearing a long skirt or dress), but more often this category was linked to women displaying expected behaviour in the community. For instance, it was considered distasteful for women to drink or smoke in the company of others, and sexual desire or promiscuity was particularly discouraged at a societal and familial level. The latter was reflected in various cultural practices in which virginity among unmarried Zulu women and girls was celebrated. These included *uKuhlolwa kwezintombi* or virginity testing ceremonies; *uMemulo* or coming of age ceremonies; and *ilobolo* or marriage negotiations, all of which have been described in depth by others [30–34].

The desire to present the right appearance also came up in discussions around sickness, as some feared that being unwell would be misinterpreted by others. For example, one participant explained that she would rather get out of bed on days when she felt sick to prevent raising suspicion among neighbours:

Sometimes the neighbours, if they didn’t see me moving around outside, they would come here [and say] ‘Oh, we are here to check [on you].’ [They think] maybe she is not well cause she is not outside… That is why I’d usually rather [get up]. (II9)

Other participants discussed similar fears about neighbours becoming aware of their health status. As in the next example, concerns often related to disclosure of one’s HIV status, suggesting to others that behavioural expectations have not been met:

You find [a lot of people] saying, ‘I would like to get checked [for HIV] once and for all,’ and once a person has confirmed that they are sick, you see them in the health facilities. And they do not want community caregivers involved because they will go around telling people who is sick… [The CCG] is like a parent to me. She is like my parent. I am supposed to be able to tell her that I was tested and found that I am like this, and there is no reason for her to tell anyone. (P1, GI8)

One participant expressed her frustration with “Zulu culture” for prioritising the appearance of health in favour of care-seeking, following the recent deaths of her husband and eldest daughter, both of whom were the breadwinners of the household: “My husband died and I didn’t know what was the cause of death. And even my [daughter] died and I didn’t know [why] because people don’t want to disclose their [health] status, and you do not know what [is] really happening” (II8). For this participant who had recently “beaten” breast cancer and reported a good experience on ART, the answer was to take control of her own health and act as a role model for her other children: “In the days when I am sick, I don’t have to go to ask [for help] from my neighbour, from my mother-in-law. I should be the one who needs to take care of my life. I need to know about my life” (II8). While this participant was similar to other participants in that she wished to avoid the prying eyes of neighbours, she was unique in that she also rejected support from her family, preferring instead to look after herself alone. Another participant who felt comfortable sharing her HIV status in the community, described how she encouraged others to overcome concerns with appearance:

Some people who get well [following ART]… disclose openly their status. Others are afraid to disclose their status to the CCGs [to seek treatment]… I encourage them that they don’t have to worry because at the end of the day [they] will be healthy. Many people come to me for help because of my openness. (II7)

For some participants, decisions to interact with healthcare providers in relation to IPT also involved concerns with appearance. For instance, one participant described her response to IPT following symptoms of extreme hunger and tingling in the hands and feet, which she associated with starting the regimen, “I decided that I would rather stop for a week so I could see whether the pills [were] the cause or not, and in a week [the symptoms] disappeared” (II9). When asked if she went back to the clinic to discuss the issues, she giggled and replied, “No, I didn’t. No, I won’t! It’s good to tell the truth. I didn’t.” When later asked if she would warn others about her IPT experience, she responded:

It might depend how close I am with that person. But the person whom I’m not close that much, I can’t talk about the side effects because I might scare that person… I will encourage that person to take [IPT] for the full 6 months, unlike myself who defaulted, because I defaulted. (II9)

## Discussion

Ultimately what encouraged many women to take IPT was the cultural gender norm of being *uMakoti*, an isiZulu word that directly translates as “the bride” or “the wife” (the prefix ‘u’ indicating the definite article), but is also a term of endearment afforded to females whose behaviour indicates maturity and high moral standing according to Zulu culture [31]. As Ngwenya explained, *Makoti* (wife, singular) is not merely a title; there are “a whole range of practices” that construct the identity, including “chores she is expected to carry out” [35]. In a more nuanced way, the title *Makoti* represents the duty to be a good woman, wife, mother, and in-law. Notions of good are demonstrated through docility: the good woman is caring, obedient, and represents herself well in the public domain. The interplay of these expectations is aptly summarised in a traditional Zulu wedding song that is sung to the bride during the wedding ceremony:

*uMakoti ungowethu* / The bride is ours

*Uzosiwashela asiphekele* / She will wash and cook for us

*Sithi helele siyavuma*! / We rejoice!

                                      *-* unknown

These findings are consistent with what contemporary Zulu scholars have called *inhlonipho*, meaning ‘to show respect.’ Rudwick explained that higher status, seniority, age, and “quite often male gender” are common qualifiers for people to whom *inhlonipho* must be directed [36]. *Inhlonipho* can be displayed through the avoidance of particular words or syllables (*isiHlonipho*) that link to the names of male in-laws, lowered gaze or posture whilst speaking to those of higher authority, and right behaviour [31,32,36]. Similar to our findings, Bhana described ‘right behaviour’ as chastity, shyness, and docility, especially among rural Zulu women who offer little economic value through work outside the home [32].

As with most medicinal interventions, valuations of successful IPT implementation are determined by measures of adherence, such as how many people start and complete therapy [37]. If what is ultimately sought in IPT intervention is adherence, then the qualities of *oMakoti* seem to support a model for achieving IPT adherence–*oMakoti* are caregivers, but ultimately not care decision makers. Indeed, our data confirm that among the women using IPT, the majority exhibited *inhlonipho* by ceding IPT decision-making power to the healthcare provider. This may explain why the majority of IPT initiators in our evaluation of IPT effectiveness were women [14].

Participants’ experiences highlight several situations in which *uMakoti* may elect to relinquish control. Firstly, she may trust implicitly the expertise of the healthcare provider and her or his interpretation that she is “in the right condition” or “the results said [she] should be on these pills.” Secondly, *uMakoti* may opt to reserve questions to appear grateful and “happy that there is help which is coming.” Thirdly, *uMakoti* may feel pressured to demonstrate *inhlonipho* for fear she may be singled out and reprimanded through health delay or exclusion for “asking too many questions” or “knowing too much.” The different intentions behind these demonstrations of *inhlonipho* highlight the circumstances in which a good *Makoti* does not necessarily equate to a good patient by Western biomedical standards–sometimes expressions of *inhlonipho* are merely performance. As Rudwick and Shange asserted, “the *hlonipha* (sic) framework is based on the idea that one must, by all means, avoid *appearing* disrespectful” [31] (our emphasis).

In the biomedical paradigm, any deviation from a medical regimen without consulting the healthcare provider is an indication that adherence has been compromised; therefore, instances in which *inhlonipho* is performed out of duty or placation rather than trust in the regimen would not conform to biomedical expectations. The practice of performance is not entirely unfamiliar to healthcare providers. Directly Observed Therapy (DOT), whereby nurses or CCGs physically witness the act of pill-taking at least five days per week, was developed by a TB clinician for the purposes of ensuring regimen compliance [38]. Similarly, health initiatives such as adherence training, which are employed in KZN upon entry into the public ART programme, are intended to educate eligible patients on the importance of pill compliance [39].

Yet, our findings do not suggest that *oMakoti* (plural form) were unaware of reasons to adhere. Decisions around initiation and discontinuation were justified with thoughtful consideration. While it may first seem perplexing that some participants would not seek help from their healthcare provider or other community members following difficulties attributed to IPT, decisions to remain silent were informed by previous experience or collective interpretations about the experience of others. In her 2013 ethnography, Beckmann described what she termed “the logic of choice” among PLWH in Tanzania, referring to the logical processes which precede healthcare decisions that may run counter to healthcare workers’ expectations [40]. Beckmann explained that practices such as performance can be “responsible” in terms of life circumstance, “but may not be considered responsible in the eyes of those trying to control [a] pandemic within a strictly biomedical framework” [40]. In essence, *uMakoti* does not feel safe to admit to the healthcare provider or others with whom she is “not that close” what she interprets as ‘disobedient’ behaviour: Don’t be like me: “I defaulted.” The very practice of identifying oneself as “defaulter” situates one in the lexicon of the biomedical framework, in which failure to complete according to a predetermined schedule results in internalisation of the decision, regardless of circumstances.

The problem is that adherence in the biomedical framework requires an openness that does not align with identity as *uMakoti*. As Kielmann and Cataldo explained, such expectations are based on a risk-based approach to preventive care in which patients are “rationally motivated to change their behaviours towards self-preservation” [41]. Community life often involves making difficult choices with minimal resources, thus quality of daily life in uMgungundlovu can take precedence to longevity. The biomedical approach fails to take into account situations in which a life worth living is dependent on functioning social relationships [40], requiring at times elements of performance in public spheres. It is not only the fear of biological manifestations of disease that determine *uMakoti*’s course of action, but also local support structures with which she identifies that may be crucial to her wellbeing. The notion of defaulter is therefore filtered through *Makoti* notions of *inhlonipho* and responses based upon more than potential physical health outcomes, but also societal expectations or fallout with ramifications to her or her household.

Not all data were consistent with women as *oMakoti*. Certainly, some women did engage more readily with healthcare providers. These women fit well with what Robins described as a new form of “responsibilised citizenship” that accounts for the ways in which illness experience in collective societies has social implications [42]. Robins described the experience of a “social death” common to South Africans newly diagnosed with HIV, which he identified as a necessary intermediate step toward a “new life” or identity as activist. Similarly, in our study, a few participants described a period in which they felt a loss of social connectedness followed by the realisation that, “I should be the one who needs to take care of my life,” in essence, rejecting the societal norm of *uMakoti*. In line with Robins’ activist model, these participants created a new life by embracing their HIV status and served as activists or sources of support for others in their community. In doing so, they chose to reinvent themselves as good patients, an identity that more closely aligns with the current biomedical notion of adherence. While we wish not to condemn this position, examples of women who fit the responsibilised citizenship model appeared infrequently in our field experience, suggesting that Robins’ theory is unlikely to represent the experience of most women in traditional Zulu communities.

### Lessons from *oMakoti*

Insofar as notions of obedience overlap with the biomedical framework, the success of IPT implementation amongst clinic-utilising women of uMgungundlovu appears to rest in large part with identity as *oMakoti*. Difficulty arises where the overlap ends: practicing *inhlonipho* at times requires the appearance of obedience, and the concept of adherence at all cost may threaten women’s social status or safety. Being *uMakoti* comes from a set of cultural expectations derived from the experiences of those who live within the community; whereas norms of ‘good patienthood’ come from a largely neoliberal paradigm of self-responsibility and gender neutrality that infiltrates from without [43]. In other words, biomedicine is born from a cultural framework established by and for the West, giving preference to certain forms of knowledge and beliefs above others. For women who identify as *oMakoti*, the practice and responsibility of *ukuHlonipha* (acting respectfully) would be much more familiar, thus explaining the less frequent occurrence of the ‘responsibilised citizen’ in our fieldwork.

We argue that successful public health interventions are best taken up when they adapt to the local culture rather than expecting the local culture to adapt. While authority may be observed in the formal clinic setting, *uMakoti* can exercise her decision-making power through ‘disobedience’ in private without disturbing “the way of life that is given.” As our data suggest, authority in the Zulu context is a fluid concept to be navigated, depending in part on levels of familiarity and the personality of the authoritarian figure. This suggests an opportunity for healthcare providers to construct less hierarchical environments, inviting *oMakoti* to interact more comfortably.

The first step is a shift from adherence-based thinking to an agency-based focus. What we can learn from *uMakoti* is that women in these settings feel comfortable providing care to those around them, yet based on Zulu notions of respect, feel less comfortable making healthcare decisions—or at least appearing as the healthcare decision-maker. The onus then falls to the healthcare provider to elicit information that can help determine the needs of potential *Makoti*, recognising that agreement does not necessarily equate to buy in. To be clear, the term ‘agency’ is not an invitation to bestow power upon a person in order to ‘correct’ behaviour, as Shefer cautioned [44]. Rather, it is an acknowledgement that the provider’s role is limited until current life complexities are ascertained. For example, what necessities must be in place for someone to be able to adhere to the IPT regimen? It is important to determine if such necessities are available to the patient at each contact, and if not, to be aware of existing support systems to which the healthcare provider can link the patient. If there are remaining barriers, the healthcare provider can communicate the potential difficulties of the regimen in light of these barriers, providing information that might help the patient to draw her own conclusions. This exercise takes place through active engagement with the patient. To assist with this process, we have included an example from Kleinman and Benson’s mini-ethnography tool [45], outlining particular steps that can help healthcare providers gain insight into the lived experience of patients who identify as *oMakoti* and work to identify any of their own pre-conceived notions of what it means to be a good patient (see S3 File).

The shift to an agency-based approach would also require healthcare providers to engage in a process of self-reflection on the ways in which their own practices may unwittingly contribute to power differentials that may impact a patient’s ability to express their preferences, despite the information provided. The harm reduction model developed by Marlatt can help to guide this process [46]. Marlatt described the harm reduction model as a means to extricate moral and medicalised expectations from care provision [46]. The first step is to recognise that biomedicine is itself a culture, one among many, and as such certain practices are taken as ‘correct’ without consideration of what is at stake for people with different life experiences–such as extreme poverty [41,43–46]. A woman who presents at clinic has by definition met the requisite of ‘patient’ in the formal healthcare system; by virtue of showing up she has demonstrated the belief that medical knowledge may benefit her and her family. She is not there to adopt a new value system; she is already working within one. The potential to assist the patient is thus limited until the healthcare provider can gain insight into the patient’s current circumstances (any combination of social, economic, political, or otherwise), which may include identification with–or rejection of–societal obligations as *uMakoti*.

Despite the pressure to meet health systems targets, initiation on and adherence to IPT ought not to be the end, but a means toward improved health outcomes in the community. Given that women are revered as caregivers, then active participation to take or not take IPT (i.e. her buy in to whichever decision) may also have ramifications on the practices of others in the household, especially children and the sick. Assessing what may affect a woman’s (or other patient’s) ability to make choices and act on them may in fact provide better insight as to how more immediate threats to health and wellness can be addressed. In other words, in agency-based practice, the ideal would be that the healthcare provider and patient work together to establish the best plan for her, and what might be altered if her circumstances change. If IPT is among her options, discuss the consequences of taking IPT, the consequences of starting and stopping IPT, and the consequences of not initiating at this time–not only the physical consequences, but also the social, economic, and emotional implications for her, and her family and community. In this way, the culture-bound moral valuations are left out of the equation, and a woman can coexist as patient and *Makoti* without the additional pressure and expectation of being ‘good’ at both.

## Conclusion

Our ethnography on IPT acceptability among Zulu women in uMgungundlovu District suggests that those who identify with *uMakoti* culture may be more likely to accept IPT on the basis of the authority position of the healthcare provider rather than personal beliefs. Under such circumstances, IPT uptake may not always equate to buy in, and may not meet the expectation of the healthcare provider in terms of adherence. Rather than interpreting such instances as a lack of commitment on the patient’s behalf, we suggest that it stems from a misalignment of value systems that may unwittingly encourage deception. Other culture-based approaches to health and wellbeing are not inferior to, and often intersect with, biomedicine. The point where they meet provides an opportunity for deeper engagement. Utilising methods to reduce authoritative influence and engage in active dialogue with patients can encourage *oMakoti* to feel heard and understood, creating a safe and informed environment that promotes patient agency instead of adherence at all cost.

## Supporting information

S1 File Group interview guide. (DOCX)

S2 File Individual interview guide. (DOCX)

S3 File Kleinman and Benson’s mini-ethnography tool [29] adapted to the context of *uMakoti*. (DOCX)
