# Supplementary material for: The role of agency in the implementation of Isoniazid Preventive Therapy (IPT): Lessons from oMakoti in uMgungundlovu District, South Africa
Source: PLoS One. 2018 Mar 7;13(3):e0193571. doi: 10.1371/journal.pone.0193571 (PMC5841771; doi:10.1371/journal.pone.0193571)
Supplement: S1 File — (DOCX) [file pone.0193571.s001.docx]

**Group interview guide**

1. Kusho ukuthini uku ``gula’’ (*What does it mean to be “sick”*)?
2. Kusho ukuthini uku ``phila” (*What does it mean to be “healthy”*)?
3. Ubani onakekela ogulayo ekhaya lakho? Emphakathini wangakini (*Who looks after the sick in your household? Your community*)?
4. Yini isifo sofuba (TB) (*What is TB*)?
5. Yini efika engqondweni uma ucabanga ngomuntu ophethwe isfiuba (TB) (*What comes to mind when you think of a person sick with TB)*?
6. Kunesikhathi esingakanani iTB iyinkinga emphakathini wakho (*How long has TB been a problem in your community*)?
7. Kungani iTB yande kangaka eNingizimu Africa (South Africa)? Emphakathini wakho (*Why is TB so common in South Africa? Your community*)?
8. Abantu bathi iTB iqhamukaphi (*Where do people say TB comes from*)?
9. Iziphi izindlela zokuvikela iTB (*What are ways to avoid TB*)?
10. Umuntu wazi kanjani ukuthi uneTB (*How would one know that they have TB*)?
11. Imaphi amakhambi asetshenziswa emakhaya okwelapha izimpawu zeTB (*What are home remedies for treatment of TB symptoms*)?
12. Uma amakhambi asekhaya engasasebenzi, umuntu uyaphi ukuthola ukulashwa? Ibe isilapheka kanjani (*If home remedies don’t work, where would one go next for treatment? How would it be treated*)?
13. Uma lokho kungabanga impumelelo, umuntu ube eseyaphi ngokulandelayo (*If that were unsuccessful, where would one go next*)?
14. Abantu bathini ngokulashwa kweTB emtholampilo nasezibhedlela (*What do people say about TB treatment in clinic or hospital*)?
15. Ingabe ukuwezwa ngeINH (*Have you heard of INH*)?
16. Wazini ngayo (*What do you know about it*)?
17. Ukhona omaziyo okewaba kuINH? Bathini ngayo? (*Do you know anyone who has been on INH? What do they say about it*)?
18. Ingabe wake wanikezwa i-INH (*Have you ever been offered INH*)?
19. Ingabe wayithatha? Kungani? (*Would you take it? Why/why not*)?
20. Iziphi ezinye izindlela zokwelapha “iTB elele” (*What are other ways to treat “sleeping TB”*)?
