# Supplementary material for: The role of agency in the implementation of Isoniazid Preventive Therapy (IPT): Lessons from oMakoti in uMgungundlovu District, South Africa
Source: PLoS One. 2018 Mar 7;13(3):e0193571. doi: 10.1371/journal.pone.0193571 (PMC5841771; doi:10.1371/journal.pone.0193571)
Supplement: S2 File — (DOCX) [file pone.0193571.s002.docx]

**Individual Interview Guide**

**English**

*C=INH completion, DC=INH Discontinued, D=INH declined, A=All*

1. (A) What does it mean to you to be “sick”?
2. (A) What does it mean to you to be “healthy”?
3. (A) What do you normally do when you feel sick?
4. (A) Who looks after the sick in your household? Your community?
5. (A) At what point do you see a physician or nurse at the local clinic? Hospital? (Prompt re: izangoma and izinyanga *local healers*)
6. (A) Think back to your last visit to the clinic. Can you tell me about it? (probe re: issues of confidentiality)
7. (A) What do you know about INH? (*Show INH pill to help remind participant*)
8. (A) Thinking back to when you were offered INH, who first offered it to you? What did they say about it? (probe confidentiality)
9. (A) Did you have any questions about INH? Did you ask them? Tell me more about that.
10. (A) Why did you decide to use/not use INH? Were there any other reasons?

10.1 (C, DC) Do you recall when you first started INH how it made you feel? Did you notice any changes over time? (Prompt with side effect examples if needed)

10.2 (C, DC) How did you get your INH? Where did you get your INH from? How did you remember to take it? (Prompt with pillbox/ help from child/family member)

10.3 (C, DC) Did you have any unanswered concerns about INH while you were on it?

10.4 (C, DC) Is there anything that you can think of that would have improved your experience on INH?

10.5 (DC) Why did you stop taking INH? Were there other reasons that made you stop taking INH?

11. (A) Do you know anyone else that has taken INH? What was their experience like?

1. What do other people say about INH in your community? (Prompt with chief, sangoma, elders, religious leaders if needed)
2. (A) What do you think about INH now?
3. (A) Would you recommend INH to other people? Why/Why not?

**isiZulu**

C=oseqedile iINH, DC=Ongaqedanga iINH, D=Oyenqabile iINH, A=Konke

1. (A) Kuchaza ukuthini “ukughula”?
2. (A) Kuchaza ukuthini “ukuphila”?
3. (A) Yini ovamisile ukuyenza umaughula?
4. (A) Ubani ovamisile ukuthi anakekele oghulayo ekhaya ? emphakathini wenu?
5. (A) kufika nini la nibona khona ukuthi sekufanele nibonane noDokotela noma onesi clinic yangakini? isibhedlela sona? (mukhumbuze ngezangoma nezinyanga abaholi bomuphakathi )
6. (A) Awuke ucabange ujule mhlazane ugcina ukuya eclinic.ungangixoxela ngakho? (kuyifinhlo yethu sobabili)
7. (A) Wazini ngokuphathwa iINH? (veza iphilisi lokumukhumbuza)
8. (A) Uyakhumbula mzukwane unikwa iINH, ubani owakunika kuqala? Wathini kuyena ngayo? (imfihlo yethu sobabili)
9. (A) Wabane mibuzo ngeINH? Wayibuza? Awungixoxela ngalokho
10. (A) Yini eyabanga ukuthi usebenzise/ungayisebenzisi iINH? Zazikhona ezinye izizathu?
    1. (C,DC) Uyakhumbula muzukwane uqala ukusebenzisa iINH wazizwa unjani? Kukhona ushentso ulubonayo ekuhambeni kwesikhathi? (mubalele imiphumela engemihle makudingeka )
    2. (C,DC) Uyithola kanjani iINH ?Uyitholaphi iINH yakho? Ukhumbula kanjani ukuyithatha iINH yakho? (mukhumbuze ngebhokisi lamaphilisi/usizo enganeni/ilunga lomundeni)
    3. (C,DC) Unakho ukukhathazeka okungaphenduliwe ngeINH ngesikhathi uyithatha?
    4. (C,DC) Kukhona yini into ongayichabanga ebingayi khuphula impilo yakho ngokuthatha iINH?
    5. (DC) yini eyabanga ukuthi uyeke ukuthatha iINH ?zikhona yini ezinye izizathu ezakwenza uthathe iINH?
11. (A) Kukhona yini obaziyo abake bathatha iINH? Ibaphathe kanjani bina?
12. Bathini abantu emuphakathini wakho ngeINH? (mubalele Inkosi,Izangoma,Abadala,Abaholi benkolo umakufanele )
13. (A) Ucabangani ngeINH manje?
14. (A) Ungayi ungayincoma iINH kubanye abaantu? ngoba/ungeke ngobani?
