# Supplementary material for: The role of agency in the implementation of Isoniazid Preventive Therapy (IPT): Lessons from oMakoti in uMgungundlovu District, South Africa
Source: PLoS One. 2018 Mar 7;13(3):e0193571. doi: 10.1371/journal.pone.0193571 (PMC5841771; doi:10.1371/journal.pone.0193571)
Supplement: S3 File — (DOCX) [file pone.0193571.s003.docx]

**Kleinman and Benson’s mini-ethnography tool [29] adapted to the context of *uMakoti***

**1.** **Assess her identity as *uMakoti*** - Does she identify as *uMakoti*? How does she view and express *inhlonipho*? Observing her dress and body language will be helpful. For example, is she wearing a long skirt? Is her head wrapped? Does she avert her eyes, gaze downward or speak little? These features may prompt questions directly related to her experience as *oMakoti*, which may be important to acknowledge and affirm in relation to health and illness.

**2.** **Determine what is at stake** **for her** – Is she responsible for the care of others at home (e.g. children, in-laws, visitors)? Who does she consult in relation to healthcare decisions (e.g. in-laws, husband, family herbalist)? What is at stake for her and her loved ones by introducing or not introducing IPT?

**3.** **Illness narrative** – How does HIV affect her or her household in the everyday? What are other daily pressures that exist for her around illness and wellbeing? Does TB factor into her list of worries/stressors? How does the threat of TB compare to other health concerns? This exercise is not about correcting or interjecting with medical information, but rather gaining insight into her lived experience and perceptions.

**4.** **Psycho social stresses** – Building upon what is at stake, what may be the psychosocial consequences of initiating IPT, collecting IPT, responding poorly to IPT, stopping IPT, or declining IPT? Can she, in her capacity of caregiver, safely reach out to those with whom she feels close to help her take decisions on how to proceed if problems arise? How might the healthcare provider’s expectations around IPT initiation create a tension for the patient, and how might these tensions be mitigated?

**5. Influence on clinical relationships** – This step is about critical self-reflection. How do a healthcare provider’s relationships (with patients, care networks, institutions, and biomedical training) affect interactions with *oMakoti*? To what degree do the providers own biases and stereotypes affect the interactions and options offered to patients? How does the provision of care in a formal clinic setting affect perceptions of authority in a provider-patient relationship? How might gestures, eye contact, personal space, and physical positioning affect power differentials? How might your comfort level differ from hers in this regard? Can the provider challenge herself or himself to make adjustments to decrease perceived imbalance?

**6.** **Recognise your limitations.** The degree to which patients relinquish decision-making power will depend on a combination of the above factors and will differ between interactions. While these steps may help to open the conversation, they will not guarantee a patient’s forthrightness. Patience is required as rapport builds, and in many scenarios, especially among traditional rural women who are closely tied to identity as *uMakoti*, it may not even be possible to elicit open discussion at first. This may just be too far from patients’ levels of comfort. However, consistently asking more open-ended questions, using culturally appropriate terms, and remaining open to alternate explanatory models can help to shift power dynamics.
